# Changes in clavicle length in acute fractures within 3 weeks: a prospective ultrasonographic study of 59 patients

**DOI:** 10.1186/s12891-017-1842-4

**Published:** 2017-11-29

**Authors:** A. H. Thorsmark, O. M. Christensen, S. Torp-Pedersen, S. Overgaard, L. H. Frich

**Affiliations:** 1 Department of Orthopedic Surgery and Traumatology, Holbæk Hospital, 4300 Holbæk, Denmark; 2 Department of Radiology, Rigshospitalet Glostrup, 2600 Glostrup, Denmark; 3 Department of Orthopedic Surgery and Traumatology, Odense University Hospital, 5000 Odensen, Denmark; 4 Department of Clinical Research, University of Southern Denmark, 5000 Odense, Denmark

**Keywords:** Ultrasound, Length measurement, Clavicle

## Abstract

**Background:**

Within traumatology a common indication for acute surgery of fractured clavicles is bone shortening over 2 cm. This indication is among but a few indications; which are recommended to be treated operatively within the very first weeks after a fracture. Theoretically clavicle fractures could become less shortened over time due to decreasing muscle pull. If this reduced shortening does indeed happen, some patients with initial bone shortening over 2 cm could perhaps be treated conservatively? However, it is unknown what happens to the length of the clavicle within the first weeks after a fracture. The aim of this study was to investigate if the length of the fresh fractured clavicles changes within the first three weeks.

**Methods:**

Rested length measurements using navigation ultrasound were done on 59 patients with a fractured clavicle. Measurements were performed within the first three weeks after a lateral or mid-clavicular fracture. The inclusion period was from March 2014 to February 2016. Median age was 40 years and age range was 18–81 years. The data was analyzed using mixed linear models.

**Results:**

The clavicle length showed no change within the first three weeks after fracture (*p* = 0.24).

**Conclusion:**

Fractured clavicles retain their length for the first three weeks.

## Background

Clavicular fractures comprise approximately 2–4% of all fractures [1]. Although often treated non-operatively with good results; several relative operative indications exist. One of these is midclavicular fractures with bone shortening of over 2 cm [2]. These fractures should be offered operative treatment within the first weeks after a fracture [3, 4]. However, it is theoretically possible that the bone becomes less shortened within the first weeks after a fracture; and an operation ultimately could be avoided. It is to date unknown if the clavicular bone actually returns to some of its former length due to decreased muscle pull. Should the bone become less shortened; it could make sense not to operate in the acute setting. If the fractures retains its shortening this postponement could result in fracture malunion and increase the risk of perioperative complications.

The authors decided to investigate if this clinical problem could be solved by measuring length changes of the clavicle within the first weeks are a fracture. The solution using existing conventional methods was limited due to the risk of bias from radiographic magnification [5]. It was hypothesized that a novel ultrasound modality, navigation ultrasound (NavUS), could be a possible candidate, as it had been previously validated to provide the required accurate length measurements [6]. The research question was; is the horizontal length of clavicle fractures stable within the first three weeks? Should the study find that the bone becomes less shortened, this could be an argument for a revision of the current status of bone shortening as a subacute operative indication and ultimately lead to more patients being treated non-operatively.

## Ethical considerations

The local ethical committee of Region Zealand, Denmark (reference number REG-67-2013) as well as the National Danish Data Registry approved the study. The study was reported to clinicaltrials.gov with the identifier NCT02089282. All participants gave their written and oral consent upon entering the study.

## Methods

The study was based on the STROBE statement [7] and was a prospective study of fresh clavicular fractures recruited from two regional hospitals within the first nine days after a fracture. The inclusion criteria were all patients who sustained a displaced/undisplaced medial, mid and lateral clavicle fracture from March 2014 to march 2016. The exclusion criteria were patients with bilateral clavicle fractures and/or prior operations involving the clavicle as well as malignant diagnoses.

The following definitions were used: 1) A medial fracture was located in the medial third, a midshaft fracture in the mid-third and a lateral fracture in the lateral third [8]. 2) A displaced fracture was defined as having a displacement of one bone width or more.

Patients were found by screening all clavicle radiographs during the inclusion period. If a eligible clavicle fracture was found the patient was contacted. Oral and written consent was gathered from the patients before they entered the study. At each session, the patients’ injured and uninjured clavicle length was measured using NavUS. Clavicular length was measured twice in both the injured and uninjured side. Patients were included within 1–9 days after sustaining a clavicular fracture with one or two follow-up sessions 20–26 days after fracture.

The NavUS system on the General Electric Loqic E9 ultrasound scanner was used. The program for musculoskeletal measurement (“msk” musculoskeletal, 14 MHz) was used along with the ML6–15 probe. The NavUS system itself consists of a bracket for the ultrasound probe with two attached sensors and a stationary transmitter. The transmitter generates a magnetic field which the sensors utilize to determine their exact spatial position. The magnet was placed opposite the side in question e.g. left to right, right to left. A distance of approximately 15 cm was kept from the shoulder. Calibration of the NavUS was done after each measurement with probe and sensors kept still on the sternum and with sensors facing away from the magnet.

The following procedures were followed.

Positioning: Patients were sitting in a wooden chair (non-metal in order to avoid interference with the magnetic field) with arms along their side. The participants were instructed to sit immobile during measurements.

Measurement: The rest measurements started with marking the sternoclavicular joint. The scanner was set in a perpendicular position to the sternoclavicular joint. Once this point was marked, the probe was moved the acromioclavicular joint while the participant remained still. A second point was marked on the acromioclavicular joint with the probe in a 30° sagittal/horizontal angle. The machine reported the distance between the two points as the resting clavicle length.

## Statistical methods

Statistical analysis was performed with STATA software (version 13.1; STATACORP, College Station, Texas). A power calculation had been made before the study with a power of 80% and an alpha of 0.05. The needed patients were 63 patients. The local ethical committee recommended that a further seven patients should be included for a total of 70 patients. Standard descriptive statistics were done for fracture type, age and gender. Mixed linear model regression analysis was used after the recommendation from an out of study statistician as multiple measurements were done at various time points after the fracture. Furthermore the use of mixed linear models allowed to adjust for gender and left/right length differences on the individual level. The length measurement performed on injured clavicles was used as the continuous response variable. Time was used as a continuous variable. Explanatory variables (covariates) were type of fracture, displaced fracture y/n, side and gender. These were chosen on their apparent connection to the pre-fracture length of the clavicle. During the model building phase the interaction terms of displacement versus days was added into the model as it was found to be statistically significant for the fit of the model.

## Results

**Fig. 1:**
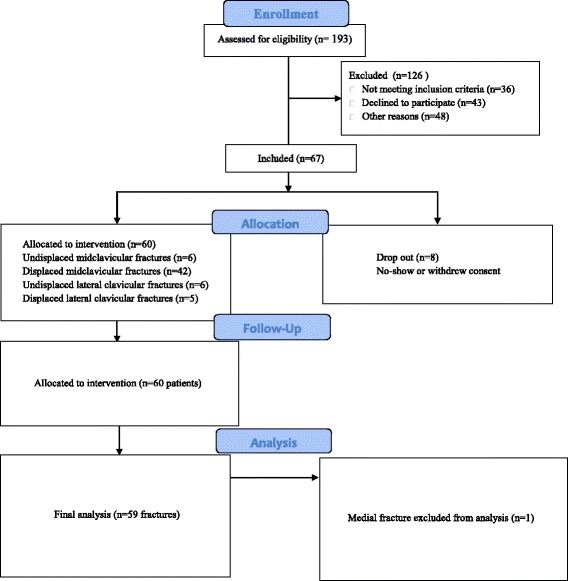
Enrollment flowchart

Fifty-nine patients who had a fracture during the period March 2014 to February 2016 were included. This number included a total of 42 male patients and 17 female patients with the median age of 40 and age range of 18–81 years. The inclusion flow-chart is shown in Fig. 1. In total, 59 patients with displaced or non-displaced mid- or lateral fractures were included. A total of 302 measurements were done with 52 missing measurements due to non-attendance and skipped appointments etc. The baseline characteristics of the patients are shown in Table 1.

**Table 1:** Base line clavicular lengths

	Mean length SD
Male	
Normal (uninjured) (*n* = 42)	152.4 mm (SD:10.5 mm)
Midclavicular displaced (*n* = 30)	145.6 mm (SD: 13.2 mm)
Lateral displaced (*n* = 4)	148.1 mm (SD: 7.6 mm)
Female	
Normal (uninjured) (*n* = 17)	138.2 mm (SD: 9.0 mm)
Midclavicular displaced (*n* = 12)	127.9 mm (SD: 7.5 mm)
Lateral displaced (*n* = 2)	123.9 mm (SD: 9.6 mm)

## Horizontal length changes within 3 weeks

The coefficient describing the length changes over the first three weeks after the fracture found a change in length of 0.1 mm which statistically insignificant (SE.: 0.1 mm; CI: −0.4 mm to 0.1 mm; *p* = 0.24). This was true for displaced fractures as well as for the interaction terms between solely displaced fractures and time which also showed a coefficient of 0 mm (SE.: 0.1 mm; CI: −0.2 mm to 0.4 mm; *p* = 0.54). Specification of data is shown in Table 2.

**Table 2:** Clavicular fracture length stability within three weeks

Length	Coefficient	Std. Err.	z	*P* > z	[95% Conf.	Interval]
Days	−0.1 mm	0.1 mm	−1.16	0.25	−.4 mm	.1 mm
Female vs. Male	18 mm	3 mm	5.59	0.000	11 mm	24 mm
Right vs. Left	0 mm	3 mm	−0.15	0.88	−6 mm	5 mm
Displaced	−9 mm	4 mm	−2.19	0.03	−16 mm	0 mm
Mid vs. Lateral	1 mm	4 mm	0.38	0.70	−6 mm	8 mm
Interaction displaced/time						
Displaced over time	0 mm	0.1 mm	0.61	0.54	−0.2 mm	0.4 mm
Length constant	137 mm	5 mm	28.14	0.000	127 mm	147 mm

## Discussion

It was found that the length of fractured clavicles did not change within the first three weeks. The ultrasound modality, NavUS, has been proven to have the ability to achieve accuracy comparable to 3D rendered CT-scans for clavicular length measurements [6]. The results are therefore reliable.

Clavicular bone shortening is one of very few indications for referral to subacute surgery as the majority of patients today are operated months later in their recovery mainly due to non-union of the fracture [9]. The fact that the fractured bone remains the same length during the first three weeks may be used to advocate for continued operation in an acute setting when a clinically significant bone shortening is present. However, the indication itself is continuously being scientifically debated [10–12] and consequently it is not all traumatological departments which follow the described paradigm. Radiographically measured bone shortening still remains a mainstay indication for surgery at least among the majority of Scandinavian traumatologists [13] since bone shortening as shown in recent survey as multiple studies have shown bone shortening to be associated with poor patient outcome [3, 4, 14].

In continuation, as it is not the bone itself which returns to its normal length; the results indicate that there may be other factors that are the cause for the shoulder to apparently return to its normal appearance. These factors could be decreasing muscle pull or reorientation of the scapula. For the time being no biomechanical study exists which describes what happens to the shoulder girdle within the first weeks and could be an interesting field for future research.

Limitations of this study include use of a technology that has not, apart from a validation study, been used in this setting before. The authors are confident of the method since it has a reliability that rivals 3D rendered CT-scans and a validity above conventional radiographs [6]. The use of a novel ultrasound method was seen as the only option to avoid the radiographic magnification of x-rays without using potentially harmful CT-scans, as patients would have needed upwards of six CT-scans to gather similar data. The final study included 59 patients which were four short of the original power calculation and eleven patients short of the number recommended by the local ethical committee. However, as the results did not show any length changes over time it does not seem likely that an additional four patients would have had any impact. Another limitation is that the data do not include any grading of the visual appearance of the shoulder; therefore, the results might be inconclusive simply because none of the patient’s’ shoulders were affected enough to show any change. No validated measure for the severity of shoulder appearance exists; consequently, it would not have been possible to avoid rater bias if we had graded the visual appearance. The authors deliberately avoided quantifying bone shortening using the side difference method which has been previously criticized for not being valid as this method relies on bilateral symmetry of the clavicles [12, 15, 16]. Other measures of bone shortening exist but a previous validation study have not shown them to be reliable [17]. The implications of this lack of reliable measurement methods to quantify bone shortening are beyond the scope of the current study. This also relates to the direct clinical interpretation of this study as the measurement method used is in this study does not have the radiographic magnification bias of upwards 20% [5]. An effort to reproduce the results of this study using conventional radiographs could therefore be difficult.Other limitations include the occurrence of outliers amongst the measurements. The statistics used do not fully display the variations between any two measurements but these measurements could be up to five millimeters. This disagreement was caused by natural measurement variation. The use of linear mixed models regression minimizes the effect as it draws the coefficient using the principle of the line of best fit thereby avoiding these. A similar situation was seen because of missing data from non-attending patients. This bias was also minimized due to regression analyzes.

A final limitation was that the authors only recruited approximately 30% of the total fractures of which the majority was male and the median age was 40 years (Fig. 1). This indicates that recruitment could be a confounder. It is therefore possible that results would be different in an older population as their muscles might not be able to stabilize fractures as well as younger individuals. The necessary age group spread to test for age in the regression models was not available.

The strengths of this study are that this is the first study of its kind to follow the length variations closely within the first weeks post-fracture. It is also the first study to implement ultrasound technology that provides the best possible accuracy other than high resolution CT. This study is unique since an equivalent study using CT would have meant a significant radiation dose to the participants.

## Conclusion

Clavicular length of fresh fractures is stable throughout the first three weeks after fracture when measured in neutral position. If radiographically measured midclavicular bone shortening is followed as an operative indication, there is no need to wait for the shortening of the fracture to change. However, as the relevance of clavicular bone shortening as an operative indication continues to be discussed scientifically and clinically our results may prove to be of use only if an operative paradigm is followed.

## Abbreviation

NavUS: Navigation ultrasound
